# Effect of Sour Taste on Swallowing Function of Patients With Dysphagia After Acute Ischemic Stroke: A Randomized Controlled Trial

**DOI:** 10.1002/brb3.71680

**Published:** 2026-08-25

**Authors:** Ayfer Gunes, Ozgul Erol, Sezgin Kehaya

**Affiliations:** ^1^ Department of Neurology Trakya University Medical Research and Application Centre Edirne Turkey; ^2^ Department of Medical Nursing, Balkan Campus Faculty of Health Sciences Trakya University Edirne Turkey

**Keywords:** acute stroke, dysphagia, nurse, sour taste, swallowing function

## Abstract

**Background:**

Untreated swallowing disorders lead to increased morbidity and mortality in acute stroke patients due to inadequate nutrition, dehydration, and aspiration pneumonia, resulting in prolonged hospital stays. Therefore, we aimed to evaluate the effect of early administration of a sour taste on the swallowing function of patients with post‐stroke dysphagia.

**Materials and Methods:**

This study was a randomized controlled trial, which was conducted with 95 patients with a confirmed diagnosis of ischemic stroke and dysphagia, admitted to the neurology department of a university hospital. The primary outcome of the study was swallowing function assessed using the Gugging Swallowing Screen (GUSS), while stroke severity assessed using the National Institutes of Health Stroke Scale (NIHSS) was evaluated as a secondary outcome. The intervention group (*n* = 47) received 4 mL of room temperature lemon juice, while the control group (*n* = 48) received 4 mL of room temperature water before breakfast, lunch, and dinner for a duration of 7 days. The Standard Swallowing Test, Gugging Swallowing Screen, and National Institutes of Health Stroke Scale were applied. Swallowing function and stroke severity were assessed at the initial assessment, at the end of the seventh and 30th days. A *p*‐value <0.05 was found as statistically significant.

**Results:**

Patients in the intervention group demonstrated significantly greater improvement in swallowing function compared with the control group on day 7 and day 30 assessments. The between‐group difference in total GUSS scores at day 30 demonstrated a large effect size (*r* = 0.54, *p* < 0.001). No statistically significant difference between groups was observed in NIHSS scores during follow‐up (all *p* > 0.05). In both groups, swallowing function improved as stroke severity decreased.

**Conclusions:**

Administration of a sour taste during the early period of acute ischemic stroke had a beneficial effect on swallowing function in patients with dysphagia. Sour taste stimulation may be considered a practical, feasible, and complementary sensory intervention in post‐stroke dysphagia management.

**Trial Registration:**

The study was registered at ClinicalTrials.gov (NCT07400822)

## Graphical abstract

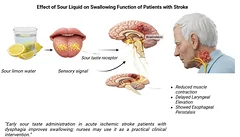

## 1 Introduction

Stroke is a neurologic disorder affecting 15 million people worldwide annually; of these, 5 million die and 5 million become permanently disabled, maintaining burden, on family and the public (Feigin et al. 2025). The prevalence of dysphagia after stroke varies widely, ranging from 8.1% to 80%, depending on the cause of the stroke, the patient's age, and environmental factors (Lu et al. 2025). Swallowing is a complex mechanism that requires the coordination of multiple muscle groups to transport food or liquid from the mouth to the stomach while minimizing residue (Zhang et al. 2022; Kang et al. 2018). Dysphagia is defined as difficulty swallowing food normally because of movement disorders in the organs involved in swallowing, which increases the risk of food entering the airway. Common signs and symptoms of dysphagia include slow swallowing speed, coughing or choking during swallowing, and a wet‐hoarse voice quality after swallowing (Zhang et al. 2022; Kim et al. 2024).

Post‐stroke dysphagia can lead to serious complications, including aspiration pneumonia, malnutrition, and dehydration, and it significantly affects the rehabilitation process of stroke patients (Xu and Zhan 2025). Management of dysphagia involves rehabilitation and therapeutic interventions aimed at minimizing aspiration‐related complications and maximizing oral intake (Peña‐Chávez et al. 2023). One such intervention is sensory stimulation. Taste is an important oral sensory stimulus that can lower the swallowing threshold (Nagano et al. 2022; Loret 2015) Sour taste, in particular, has been shown to trigger swallowing more effectively than other stimuli and to produce stronger swallows (Dafiah and Swapna 2020). Sour taste functions as a peripheral gustatory stimulus that may facilitate activation of the central swallowing network. Gustatory afferent inputs are primarily transmitted through cranial nerves VII (facial), IX (glossopharyngeal), and X (vagus) to the nucleus tractus solitarius (NTS) in the brainstem, which plays a central role in swallowing regulation, airway protection, and sensorimotor integration (Andresen and Kunze, 1994; Jean, 2001). Activation of these pathways may facilitate triggering of the swallowing reflex by lowering the swallowing threshold and enhancing coordination of swallowing‐related motor responses (Jean, 2001; Mulheren et al. 2016). In addition to reflexive swallowing control, the NTS interacts with supranuclear and cortical swallowing networks, suggesting that peripheral gustatory stimulation may support functional activation of the central swallowing system after stroke (Rinaman 2011; Simonyan and Horwitz 2011).

Lemon juice (pH = 2.8) was selected as the sour stimulus because it is a natural, inexpensive, easily accessible, and clinically applicable source of citric acid that provides strong gustatory stimulation. Previous dysphagia studies have demonstrated that acidic and sour stimuli, particularly citric acid‐containing liquids, may facilitate swallowing initiation and improve swallowing safety in patients with neurogenic and post‐stroke dysphagia (Pelletier and Lawless 2003; Cola et al. 2010; Gatto et al. 2013). In addition, the acidity level of lemon juice allows delivery of intense sour stimulation with a small administration volume, which may be advantageous for minimizing aspiration risk during bedside application (Pelletier and Lawless 2003; Cola et al. 2010).

Although thin liquids may increase aspiration risk in some dysphagic patients, sour stimulation protocols are generally administered in controlled and minimal volumes to reduce potential airway compromise while maximizing sensory input. In the present study, only 4 mL of lemon juice was administered under physician supervision after bedside dysphagia screening, and patients were closely monitored during administration. The intervention was designed as a sensory stimulation approach rather than nutritional oral feeding. Previous studies have also suggested that sour stimuli may improve swallowing safety by facilitating earlier swallowing initiation and enhancing airway protective responses (Mulheren et al. 2016; Cola et al. 2010). The liquid consistency and administration procedure were determined after bedside swallowing screening and were applied only under clinical supervision within the routine dysphagia care pathway (Peña‐Chávez et al. 2023).

Early diagnosis and management of post‐stroke dysphagia are essential for preventing complications and improving quality of life. Speech‐language pathologists are the primary specialists responsible for comprehensive dysphagia assessment, determination of safe swallowing strategies, and diet modification, whereas nurses play an important supportive role in early screening, monitoring swallowing‐related symptoms, implementing prescribed interventions, and ensuring continuity of care (Peña‐Chávez et al. 2023; Zhang et al. 2022). Therefore, the present intervention was conducted within an interdisciplinary dysphagia management approach under physician supervision and in accordance with standard dysphagia care protocols. Sour taste stimulation was not intended to replace standard dysphagia therapy but rather to serve as a complementary sensory stimulation method alongside routine clinical management (Wang et al. 2025).

The study was designed to evaluate the effect of early administration of sour taste on the swallowing function of patients who developed dysphagia after stroke. The primary objective of the study was to evaluate the effect of sour taste stimulation on swallowing function measured by GUSS scores in patients with post‐stroke dysphagia. The secondary objective was to examine changes in stroke severity assessed by NIHSS scores during follow‐up. It was hypothesized that administration of a sour taste would increase GUSS swallowing scores by facilitating swallowing function and reducing the severity of dysphagia during the early post‐stroke period.

## 2 Methods

### 2.1 Study Design

This study was designed as a parallel‐group, randomized controlled superiority trial with a 1:1 allocation ratio. It was conducted in the neurology clinic of a university hospital between July 15, 2021, and November 20, 2022. Patients met the following inclusion criteria: (1) hospitalized with a confirmed diagnosis of ischemic stroke, (2) within the first 72 h after diagnosis, (3) developed dysphagia, (4) who could understand and cooperate, (5) voluntarily signed an informed consent. Exclusion criteria are: (1) patients with cerebral hemorrhage or other neurologic disorders, (2) patients with severe stroke and high NIHSS scores, (3) who were likely to experience severe dysphagia. Patients considered likely to experience severe dysphagia were identified during the initial bedside Standard Swallowing Test. Patients who were unable to manage saliva, demonstrated drooling, were unable to complete the initial 5 mL water swallowing step, could not follow verbal instructions, or presented with lethargy/drowsiness were considered to have severe dysphagia and were not included in the study. In addition, patients with Glasgow Coma Scale scores below 8 during the acute period were transferred to intensive care and were not enrolled. The random allocation sequence was generated by the researchers using a computer‐based randomization table with simple randomization procedures. No stratification procedure was applied. Participants were assigned to the intervention and control groups according to a 1:1 allocation sequence. Allocation concealment was maintained using sequentially numbered opaque sealed envelopes prepared before participant enrollment. Participants were allocated to the intervention and control group using a randomization table (*n* = 99). This study was registered in a clinical trials database (NCT07400822) and conducted in accordance with the CONSORT (Consolidated Standards of Reporting Trials) guidelines. CONSORT is a reporting guideline developed to improve the quality of reporting of randomized controlled trials (RCTs) and to facilitate their critical appraisal and interpretation (Moher et al. 2010).

### 2.2 Sample

All patients who met the study inclusion criteria and voluntarily agreed to participate between July 15, 2021, and November 20, 2022, were included (*n* = 99). During the study period, patients with severe dysphagia who could not complete the initial bedside swallowing screening or who required intensive care monitoring were not enrolled for patient safety reasons. Therefore, the study findings should primarily be interpreted for patients with mild to moderate post‐stroke dysphagia who were clinically stable enough to participate in bedside swallowing assessments. An a priori sample size analysis was performed using G*Power software (version 3.1). Based on previous dysphagia intervention studies reporting moderate‐to‐large intervention effects (Wang et al. 2025), an effect size of *d* = 0.60, α = 0.05, statistical power of 80%, and a 1:1 allocation ratio were assumed. Accordingly, the minimum required sample size was calculated as 90 participants (45 per group). Considering possible attrition during follow‐up, 99 participants were recruited. To determine the adequacy of the sample, the effect size determination method (*d*‐value) developed by Cohen was used. Using the data from this study, a *d*‐value of 0.907 was calculated for the difference in GUSS final evaluations between the study groups. Using the G‐power software (version 3.1) and the findings of our study, a power analysis was conducted with a confidence level of 95% (1‐α), resulting in a test power (1‐β) of 98% for the Mann–Whitney *U* test analysis. Based on these findings, it can be concluded that the research sample was sufficient.

### 2.3 Randomization and Blinding

**FIGURE 1:**
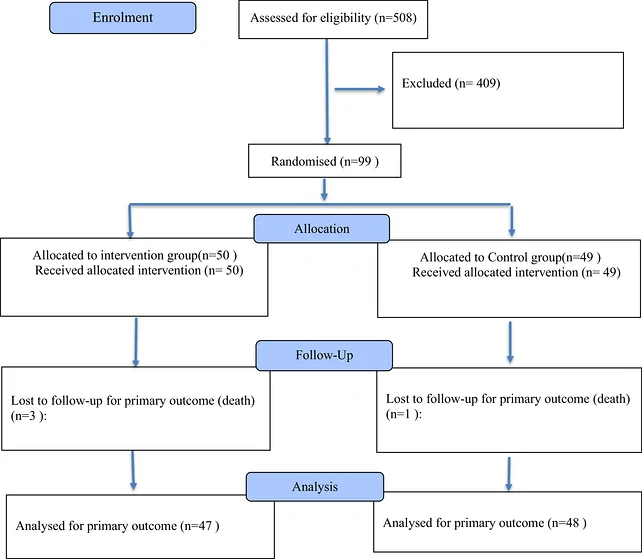
CONSORT 2025 Flow diagram.

The random allocation sequence was generated by the researchers using a computer‐based randomization table with simple randomization procedures and a 1:1 allocation ratio. No blocking or stratification methods were applied. Allocation concealment was maintained using sequentially numbered opaque sealed envelopes prepared before participant enrollment. The patients were randomly assigned to the intervention and control groups (*n* = 99) according to the randomization table (Random Sorting, Maximum Allowable % Deviation = 10%) to preserve internal validity and prevent researcher bias. A single‐blind method was used to prevent bias and evaluate the effectiveness of sour taste. The study was designed such that participants were not informed about the intervention administered to the other study group during the study period. However, because the sour taste could be recognized by participants receiving lemon juice, complete participant blinding was not fully feasible, which may have increased the risk of performance bias. Care providers administering the intervention were aware of group allocation because of the nature of the intervention. Outcome assessments using the GUSS and NIHSS were conducted according to standardized clinical evaluation procedures. Data analyses were performed after completion of data collection. During the implementation process, three patients from the intervention group and one patient from the control group were excluded from the study due to mortality. The study was planned and implemented with a total of 95 patients suitable with inclusion criteria, 47 in the intervention group and 48 in the control group (Figure 1).

### 2.4 Data Collection Tools

Data were collected using the Personal Information Form, the Gugging Swallowing Screening Test (GUSS), and the National Institutes of Health Stroke Scale (NIHSS). The primary outcome of the study was swallowing function assessed using GUSS scores, whereas the secondary outcome was stroke severity assessed using NIHSS scores. Outcome assessments were performed at baseline, day 7, and day 30.

#### 2.4.1 Personal Information Form

Based on a literature review, researchers prepared a personal information form including 21 questions about sociodemographic (e.g., age, gender, marital status, place of residence, education, occupation, monthly income, etc.) and clinical characteristics (e.g., diagnosis period, stroke subtype, affected brain region, number of lesions, stroke severity, presence of chronic illness, etc.) of patients. Clinical characteristics related to dysphagia management and swallowing safety were also monitored throughout the study period according to routine clinical care procedures.

#### 2.4.2 Standard Swallowing Test

The Standard Swallowing Test is a bedside clinical screening tool with high sensitivity for detecting aspiration risk in patients with swallowing disorders. The test evaluates speech impairment, limited tongue movement, abnormal voice quality, inadequate voluntary cough, abnormal pharyngeal sensation, and the presence of coughing or wet voice after consecutive swallowing of 5 and 50 mL of water. During the procedure, if any abnormal finding is observed while administering 5 mL of water three consecutive times, the assessment is terminated to prevent aspiration risk (Thiyagalingam et al. 2021). In the present study, the Standard Swallowing Test was used as an initial bedside screening method to identify patients with dysphagia before further evaluation with the GUSS. The test was also used to identify patients with severe dysphagia who were unable to safely complete bedside swallowing assessment procedures prior to study enrollment.

#### 2.4.3 Gugging Swallowing Screen (GUSS)

The GUSS is a simple, bedside dysphagia screening tool designed for nurses and therapists working with acute stroke patients. The tool consists of two sections: a preliminary assessment (indirect swallowing test) and a direct swallowing test.

In the preliminary assessment, swallowing is evaluated through three sequential subtests. Each subtest is scored out of five points, and completion of a five‐point section is required before proceeding to the next subtest. The direct swallowing test evaluates swallowing with different food consistencies, including liquids, semi‐solids, and solids. Evaluation criteria include the ability to swallow, presence of involuntary coughing, and drooling. Normal swallowing is scored as 20 points. Scores of 19–15 indicate mild dysphagia, 14–10 indicate moderate dysphagia, and 9–0 indicate severe dysphagia (Selg et al. 2025; Umay et al. 2019). Although GUSS was originally developed as a bedside dysphagia screening tool, previous studies have demonstrated its validity and clinical utility in monitoring swallowing status and dysphagia severity in acute stroke patients during follow‐up assessments (Lopes et al. 2019; Selg et al. 2025). Therefore, GUSS was used in the present study to evaluate changes in swallowing function over time. In the present study, GUSS scores obtained at baseline, day 7, and day 30 were used as the primary outcome measure for evaluating changes in swallowing function during follow‐up.

#### 2.4.4 National Institutes of Health Stroke Scale (NIHSS)

The NIHSS is a widely used scoring system for assessing neurological deficits in acute stroke patients. The scale is easy to administer, quick, and provides a valid measure of stroke severity (Alemseged et al. 2022; Kogan et al. 2020). The NIHSS score is defined as the sum of 15 individually assessed items and ranges from 0 to 42. Stroke severity can be categorized as follows: no stroke symptoms, 0; mild stroke, 1–4; moderate stroke, 5–15; moderate to severe stroke, 16–20; and severe stroke, 21–42 (Chalos et al. 2020). NIHSS assessments performed at baseline, day 7, and day 30 were used as the secondary outcome measure to evaluate changes in neurological status during follow‐up.

### 2.5 Applications

At the initial visit, patients with dysphagia identified through the Standard Swallowing Test underwent further evaluation using the GUSS and the NIHSS to determine swallowing function and stroke severity. The primary outcome of the study was swallowing function assessed by GUSS scores, whereas the secondary outcome was stroke severity assessed by NIHSS scores. Patients in both groups were evaluated at baseline, day 7, and day 30. Both groups received routine clinical dysphagia care throughout the study period. Standard care included upright positioning during feeding, maintaining the head of the bed at 45°–60° for at least 30 min after nasogastric feeding, positioning the head at 90° during oral intake, use of thickened liquids and texture‐modified foods for patients with mild or moderate dysphagia in collaboration with the dietitian, and implementation of an oral care protocol for all patients. Sour taste stimulation was administered as a complementary sensory intervention in addition to routine clinical care.

Fresh lemon juice used in the intervention group was prepared daily from commercially available fresh lemons obtained from the hospital food supply. Lemon juice was freshly squeezed before administration and standardized to a pH value of 2.8 at room temperature. pH measurements were performed using a calibrated digital pH meter prior to administration, and the pH value was verified during each preparation period to ensure consistency across participants. All participants in the intervention group received the same administration volume (4 mL), administration frequency, and application procedure throughout the study period.

#### 2.5.1 Control Group Application

**FIGURE 2:**
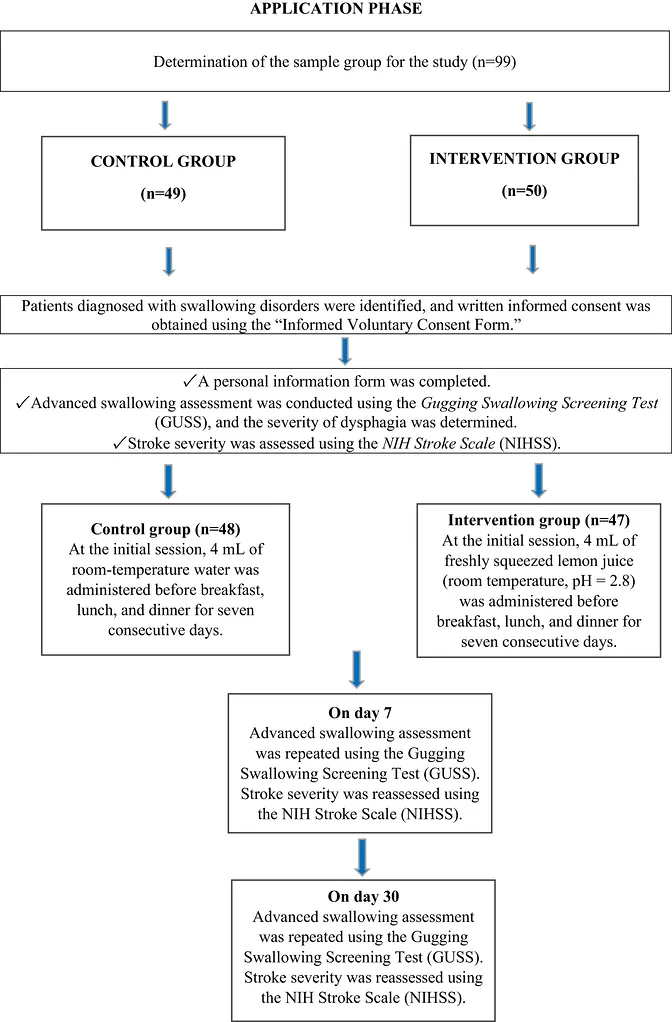
Workflow diagram.

Control group patients were administered 4 mL of room temperature water during the initial consultation under physician supervision before breakfast, lunch, and dinner. According to the GUSS screening results, patients with severe dysphagia received 4 mL of water drop by drop using a syringe over approximately 15–20 min according to patient tolerance. The administration procedure was standardized for all participants in the control group with respect to administration volume, timing, frequency, and patient positioning during application. The 4 mL volume was selected based on the protocol described by Wang et al. (2019) and was preferred as a low‐volume sensory stimulus to minimize aspiration risk while allowing controlled bedside administration. Patients with severe dysphagia who were unable to swallow expelled the administered water from the mouth as drooling. This procedure was continued for seven consecutive days (Figure 2).

#### 2.5.2 Intervention Group Application

Patients in the intervention group were administered 4 mL of freshly squeezed lemon juice (pH = 2.8) at room temperature under physician supervision before breakfast, lunch, and dinner. Fresh lemon juice was prepared daily using the same preparation procedure and standardized administration protocol for all participants in the intervention group. According to the GUSS screening results, patients with severe dysphagia received 4 mL of lemon juice drop by drop using a syringe over approximately 15–20 min according to patient tolerance. The administration procedure, patient positioning, administration frequency, and application duration were standardized throughout the intervention period. Patients with severe dysphagia who were unable to swallow expelled the administered lemon juice from the mouth as drooling. The 4 mL volume was selected based on the protocol described by Wang et al. (2019) and was preferred as a low‐volume sensory stimulus to minimize aspiration risk while providing adequate gustatory stimulation. This intervention continued for seven consecutive days (Figure 2).

#### 2.5.3 Intervention and Control Group Applications

Patients with dysphagia after acute ischemic stroke in both the intervention and control groups were evaluated at baseline, day 7, and day 30. Baseline and day 7 evaluations were conducted in the neurology clinic while patients were hospitalized. Day 30 evaluations were conducted in the neurology outpatient clinic to reassess swallowing function and stroke severity. All outcome assessments were performed using the same standardized bedside evaluation procedures and assessment tools at each follow‐up time point. Because neurological recovery may continue during the post‐stroke period, the 30‐day assessments were interpreted within the context of both spontaneous recovery and routine clinical care processes in addition to the potential effects of sensory stimulation.

Routine clinical dysphagia care at our institution was provided according to the hospital's standard stroke management protocol. Standard care included bedside swallowing assessment, individualized dietary modification (texture‐modified diets and thickened liquids when clinically indicated), appropriate nutritional and hydration support, upright positioning during oral feeding (or head‐of‐bed elevation to 30–45° for patients receiving enteral nutrition), routine oral hygiene, aspiration precautions, and regular monitoring for signs of aspiration, malnutrition, and dehydration. Enteral nutrition was provided when oral intake was considered unsafe or insufficient based on the treating clinical team's assessment.

### 2.6 Statistics

Data were analyzed using descriptive statistics, the Kolmogorov–Smirnov test, the Mann–Whitney *U* test, the Kruskal–Wallis test, the Pearson chi‐square test, the Yates continuity correction chi‐square test, Fisher's exact test, the Bonferroni test, the Independent Samples *t*‐test, the Wilcoxon signed‐rank test, Spearman correlation analysis, and the Friedman test. Effect size for the primary outcome was calculated using the r statistic derived from the Mann–Whitney *U* test. A *p*‐value <0.05 was considered statistically significant. Given the observational follow‐up period until day 30, findings related to long‐term swallowing improvement were interpreted cautiously due to the possible influence of spontaneous neurological recovery and concurrent routine clinical care.

## 3 Results

A total of 95 patients (47 in the intervention group and 48 in the control group) met the inclusion criteria for the study. During the study period, three participants in the intervention group and one participant in the control group died due to complications related to acute ischemic stroke and associated clinical conditions. No deaths were considered related to the study intervention. All remaining participants completed follow‐up assessments and were included in the final analyses (Figure 1).

In the intervention group, the mean age was 71.7 ± 11.4 years; 57.4% were women; 78.7% had primary school education; 72.3% reported income equal to expenses; 83.0% had a chronic illness; 63.8% had no previous history of ischemic stroke; 40.4% had posterior circulation infarct; and 51.1% had multiple lesions. In the control group, the mean age was 72.4 ± 11.5 years, 56.3% were men, 81.3% had primary school education, 58.3% reported income equal to expenses, 77.1% had a chronic illness, 58.3% had no previous history of ischemic stroke, 47.9% had partial anterior circulation infarct, and 52.1% had single lesions. No statistically significant differences were found between the intervention and control groups regarding demographic characteristics or stroke‐related clinical features (all p > 0.05; Table 1).

**TABLE 1:** Distribution of patients' individual characteristics and stroke‐related features.

Characteristics		Intervention group	Control group	t/χ2	p
		(*n* = 47)	(*n* = 48)		
**Age, Mean ± SD (year)**		71,7±11,4	72,4±11,5	0,323*	0,748
		***n* (%)**	***n* (%)**		
**Gender**	Female	27(57,4)	21(43,8)	1,782^‡^	0,259
	Male	20(42,6)	27(56,3)		
**Marital status**	Married	22(46,8)	32(66,7)	3,051^†^	0,081
	Single	25(53,2)	16(33,3)		
**Education status**	Illiterate	6(12,8)	2(4,2)	4,909^†^	0,179
	Primary school	37(78,7)	39(81,3)		
	High school	1(2,1)	5(10,4)		
	Bachelor	3(6,4)	2(4,2)		
**Income status**	Less than expenses	11(23,4)	17(35,4)	2,056^†^	0,358
	Equals expenses	34(72,3)	28(58,3)		
	More than expenses	2(4,3)	3(6,3)		
**Chronic illness**	Yes	39(83,0)	37(77,1)	0,213^‡^	0,644
	No	8(17,0)	11(22,9)		
**Stroke time**	Day 0	36(76,6)	33(68,8)	*3,025* ^†^	*0,220*
	1^st^ day	7(14,9)	5(10,4)		
	2^nd^ day	4(8,5)	10(20,8)		
**Prior history of ischemic stroke**	Yes	17(36,2)	20(41,7)	*0,115* ^‡^	*0,735*
	No	30(63,8)	28(58,3)		
**Stroke subtype**	TACI	5(10,6)	5(10,4)	*1,070* ^†^	*0,784*
	PACI	18(38,3)	23(47,9)		
	LACI	5(10,6)	5(10,4)		
	POCI	19(40,4)	15(31,3)		
**Area affected by stroke**	Right hemisphere	20(42,6)	30(62,5)	*4,414* ^†^	*0,353*
	Left hemisphere	19(40,4)	14(29,2)		
	Brainstem	4(8,5)	2(4,2)		
	Cerebellum	1(2,1)	1(2,1)		
	Multiple	3(6,4)	1(2,1)		
**Number of lesions**	Single	23(48,9)	25(52,1)	*0,010* ^‡^	*0,919*
	Multiple	24(51,1)	23(47,9)		

*p* > 0,05; *t: Independent Sample t‐test; **χ2 =** Chi‐Square Tests (†:Pearson Chi‐Square Test, ‡: Yates Chi‐Square Test, §: Fisher's Exact Test); Abbreviations: BMI, body mass index; LACI, Lacunar İnfarct; PACI: Partial Anterior Circulation İnfarct;; POCI: Posterior Circulation İnfarct; SD: Standard deviation; TACI: Total Anterior Circulation İnfarct.

### 3.1 Primary Outcome: Swallowing Function (GUSS)

At baseline, no statistically significant difference was found between the intervention and control groups regarding dysphagia severity according to GUSS swallowing categories (χ^2^ = 1.188, p = 0.552). However, statistically significant differences were observed on day 7 (χ^2^ = 15.980, p = 0.001) and day 30 (χ^2^ = 16.502, p = 0.001), indicating lower dysphagia severity in the intervention group (Table 2).

**TABLE 2:** Risk ratios of Swallowing Disorder and Comparison of Mean GUSS Scores between intervention and control groups.

		Intervention group (*n* = 47)	Control group (*n* = 48)		
**Follow up**	**GUSS score**	***n* (%)**	***n* (%)**	***χ2***	***p***
**İnitial assessment**	Severe dysphagia	14(29,8)	10(20,8)	*1,188*	*0,552*
	Moderate dysphagia	21(44,7)	26(54,2)		
	Mild dysphagia	12(25,5)	12(25,0)		
**Seventh day**	Severe dysphagia	5(10,6)	7(14,6)	***15,980***	***0,001*** ^†^
	Moderate dysphagia	7(14,9)	24(50,0)		
	Mild dysphagia	32(68,1)	16(33,3)		
	No dysphagia	3(6,4)	1(2,1)		
**Thirtieth day**	Severe dysphagia	3(6,4)	4(8,3)	***16,502***	***0,001*** ^†^
	Moderate dysphagia	5(10,6)	18(37,5)		
	Mild dysphagia	25(53,2)	24(50,0)		
	No dysphagia	14(29,8)	2(4,2)		
**GUSS**	**Measurement**	**Mean ± SD**	**Mean ± SD**	***Z***	***p***
**Indirect**	Initial assessment^a^	3,43 ± 0,77	3,38 ± 0,99	*−0,230*	*0,818*
	Seventh day^b^	4,19 ± 0,71	3,69 ± 0,95	***−2,780***	***0,005*** ^||^
	Thirtieth day^c^	4,40 ± 0,74	4,08 ± 0,99	***−2,361***	***0,018*** ^||^
	***χ2***	***43,400***	***25,920***		
	***p***	***<0,001*** ^||^	***<0,001*** ^||^		
	***Difference*** ¶	*a<b<c*	*a<b<c*		
**Direct**	Initial assessment^a^	7,62±3,69	7,67±3,73	*−0,157*	*0,875*
	Seventh day^b^	10,64 ± 4,15	9,10 ± 3,34	***−3,265***	***0,001*** ^||^
	Thirtieth day^c^	13,45 ± 2,18	10,60 ± 2,86	***−5,485***	***<0,001*** ^||^
	***χ2***	***70,257***	***29,219***		
	***p***	***<0,001*** ^||^	***<0,001*** ^||^		
	***Difference*** ¶	*a<b<c*	*a<b<c*		
**Total**	Initial assessment^a^	11,04 ± 4,25	11,02 ± 4,50	*−0,075*	*0,940*
	Seventh day^b^	15,04 ± 4,41	12,54 ± 4,36	***−3,692***	***<0,001*** ^||^
	Thirtieth day^c^	17,85 ± 2,79	14,31 ± 3,90	***−5,228***	***<0,001*** ^||^
	***χ2***	***74,115***	***33,841***		
	***p***	***<0,001*** ^||^	***<0,001*** ^||^		
	***Difference***	*a<b<c*	*a<b<c*		

^†^ :p < 0,05; ***χ2*** *=* Pearson Chi‐Square Test;

^||^ :p < 0,05; ***χ2 =*** Friedman test; ¶: Wilcoxon signed‐rank test; **Z**:Mann–Whitney *U* test;

Abbreviation: GUSS, Gugging Swallowing Screen; SD, Standard deviation.

No statistically significant differences were observed between the groups in mean GUSS indirect, direct, or total scores at baseline (Indirect: Z = −0.230, p = 0.818; Direct: Z = −0.157, p = 0.875; Total: Z = −0.075, p = 0.940). However, the intervention group demonstrated significantly higher GUSS indirect scores on day 7 (Z = −2.780, p = 0.005, *r* = 0.29) and day 30 (Z = −2.361, p = 0.018, *r* = 0.24) compared with the control group. Similarly, GUSS direct scores were significantly higher in the intervention group on day 7 (Z = −3.265, p = 0.001, *r* = 0.34) and day 30 (Z = −5.485, *p* < 0.001, *r* = 0.56). Total GUSS scores were also significantly higher in the intervention group on day 7 (Z = −3.692, p < 0.001, *r* = 0.38) and day 30 (Z = −5.228, p < 0.001, *r* = 0.54), with a large effect size observed for the day 30 comparison. The mean between‐group difference in total GUSS scores was 2.50 points on day 7 and 3.54 points on day 30 in favor of the intervention group.

Within‐group analyses demonstrated significant improvements over time in GUSS indirect, direct, and total scores in both groups according to Friedman test results (intervention group: χ^2^ = 43.400, χ^2^ = 70.257, χ^2^ = 74.115, respectively, all p < 0.001; control group: χ^2^ = 25.920, χ^2^ = 29.219, χ^2^ = 3 3.841, respectively, all p < 0.001) (Table 2).

### 3.2 Secondary Outcome: Stroke Severity (NIHSS)

No statistically significant differences were observed between the intervention and control groups in NIHSS scores at baseline (Z = −0.930, p = 0.352), day 7 (Z = −0.150, p = 0.881), or day 30 (Z = −0.049, p = 0.961). However, within‐group analyses demonstrated significant reductions in NIHSS scores over time according to Friedman test results in both the intervention group (χ^2^ = 46.052, p < 0.001) and the control group (χ^2^ = 26.510, p < 0.001), indicating decreased stroke severity during follow‐up (Table 3).

**TABLE 3:** Comparison of mean NIH scores between intervention and control groups.

	Intervention group (*n* = 47)	Control group (*n* = 48)		
**NIHSS‐Measurement**	**Mean ± SD**	**Mean ± SD**	***Z***	***p***
İnitial assessment^a^	7,04 ± 5,25	6,00 ± 4,49	*−0,930*	*0,352*
Seventh day^b^	5,02±4,84	5,17 ± 5,10	*−0,150*	*0,881*
Thirtieth day^c^	4,09 ± 4,29	4,21± 4,76	*−0,049*	*0,961*
***χ2***	***46,052***	***26,510***		
***p***	***<0,001*** ^||^	***< 0,001*** ^||^		
***Difference***	*a<b<c*	*a<b<c*		

^||^ **:*p*** < 0,05; ***χ2*** *=* Friedman test; ¶: Wilcoxon signed‐rank test; Z: Mann–Whitney *U* test;

Abbreviations: SD, Standard deviation; NIHSS, National Institute of Health Stroke Scale.

A statistically significant negative correlation was observed between NIHSS and GUSS scores. In the intervention group, correlation coefficients were *r* = −0.418 (p = 0.003) at baseline and *r* = −0.466 (p = 0.001) at day 30, whereas the day 7 correlation was not statistically significant (*r* = −0.286, p = 0.051). In the control group, statistically significant negative correlations were observed at baseline (*r* = −0.478, p = 0.001), day 7 (*r* = −0.433, p = 0.002), and day 30 (*r* = −0.564, *p* < 0.001), indicating that swallowing function improved as stroke severity decreased (Table 4).

**TABLE 4:** Comparison of NIHSS and GUSS Scores between the intervention and control groups.

	Scale	Test	Initial assessment	Seventh day	Thirtieth day
**Intervention group**	**GUSS**	***r***	−0,418	−0,286	−0,466
	**NIHSS**	***p***	**0,003** ^††^	0,051	**0,001** ^††^
	**Scale**	**Test**	**Initial assessment**	**Seventh day**	**Thiftieth day**
**Control group**	**GUSS**	***r***	−0,478	−0,433	−0,564
	**NIHSS**	***p***	**0,001** ^††^	**0,002** ^††^	**<0,001** ^††^

^††^ :*p* < 0,05; ***r***: Spearman correlation test;

Abbreviations: GUSS, Gugging Swallowing Screen; NIHSS, National Institute of Health Stroke Scale.

### 3.3 Harms and Adverse Events

No intervention‐related serious adverse events were observed during the study period. No participants developed clinically documented aspiration pneumonia associated with the intervention protocol. Temporary coughing episodes and drooling were observed in some patients with severe dysphagia during bedside administration procedures in both groups; however, these findings were consistent with the patients’ baseline swallowing impairment and did not require discontinuation of the intervention. No oral irritation, allergic reaction, or intolerance related to lemon juice administration was reported.

## 4 Discussion

Untreated and undetected swallowing disorders lead to increased morbidity and mortality in acute stroke patients due to inadequate nutrition, dehydration, and aspiration pneumonia, resulting in prolonged hospital stays, decreased functionality, and increased long‐term care needs. Early detection of dysphagia in stroke patients improves outcomes by preventing complications such as pulmonary infections and malnutrition (Feigin et al. 2025; Smith et al. 2018; Chen et al. 2019). Consequently, early diagnosis and timely intervention for post‐stroke dysphagia are critical components of patient care management (Khedr et al. 2021; Roje‐Bedeković et al. 2020). Previous studies investigating the effect of sour taste on post‐stroke dysphagia have demonstrated its therapeutic potential (Dafiah and Swapna 2020; Abdul Wahab et al. 2011; Kang et al. 2025). This study aimed to evaluate the effect of early administration of a sour liquid on swallowing function in post‐stroke patients with dysphagia.

Patient demographic characteristics, stroke‐related characteristics, initial mean GUSS indirect and direct test scores, total GUSS scores, and mean NIHSS scores at the initial, day 7, and day 30 assessments were comparable between the intervention and control groups, confirming group homogeneity. The absence of statistically significant differences between groups ensures that the outcomes were not confounded by individual characteristics, stroke‐related features, or baseline swallowing and NIHSS scores.

Several screening tools with varying strengths and limitations have been developed to assess swallowing. The GUSS bedside swallowing screening test, a validated patient‐centered tool, demonstrates high sensitivity in identifying acute stroke patients at risk of aspiration (Ferreira et al. 2018; Lopes et al. 2019) and is the only screening tool assessing swallowing across multiple consistencies in acute stroke‐related dysphagia (John and Berger 2015; Karaca Umay et al. 2018). Comparisons with Flexible Endoscopic Evaluation of Swallowing (FEES) using the Penetration‐Aspiration Scale (PAS) have confirmed that GUSS is a valid and reliable tool for evaluating dysphagia by different healthcare professionals (Selg et al. 2025). For these reasons, the GUSS test was used in this study. However, because instrumental swallowing assessments such as videofluoroscopic swallowing study (VFSS) or FEES were not performed, objective physiological confirmation of aspiration reduction and swallowing biomechanics could not be established in the present study. Therefore, the findings should be interpreted within the context of bedside clinical swallowing assessment results.

The 7‐day and 30‐day assessments showed that the intervention group experienced significant improvements in swallowing function compared to the control group. Wang et al. (2019) reported that taste stimuli can effectively improve post‐stroke swallowing function, with regular administration producing significant benefits. Previous studies have demonstrated beneficial effects of sensory stimulation on swallowing function in post‐stroke dysphagia. Lee et al. (2012) reported that sour liquids improved swallowing timing and reduced aspiration risk in patients with swallowing disorders, whereas simultaneous sour and cold stimulation further enhanced pharyngeal transit in ischemic stroke patients. Similarly, Cola et al. (2010) demonstrated beneficial effects of combined sour and cold stimulation on swallowing physiology. However, unlike these studies, the present intervention was administered at room temperature without cold stimulation. Therefore, the observed improvements in swallowing function in the current study may primarily reflect the facilitatory effect of sour taste as a peripheral gustatory stimulus rather than thermal stimulation. In the present study, mean GUSS scores on days 7 and 30 confirmed that the intervention group had significantly lower rates of swallowing disorders, indicating a reduced risk of dysphagia. Accordingly, the hypothesis that early administration of a sour liquid improves swallowing function was supported.

Previous studies have reported similar findings. Pelletier and Lawless (2003) found that citric acid (pH: 2.7) improved swallowing in patients with neurogenic dysphagia compared to water (Pelletier and Lawless 2003). Gatto et al. (2013) demonstrated that sour taste and cold stimuli reduced oral transit time in patients with mild to moderate oropharyngeal dysphagia after ischemic stroke, assessed via videofluoroscopy (Gatto et al. 2013). Gatto et al. (2021) also reported shorter swallowing transit times and increased swallowing frequency in stroke patients receiving sour or cold sour liquids (Gatto et al. 2021). Although previous preliminary studies have suggested that acidic taste stimulation may influence neural activity associated with swallowing function (Kang et al. 2025), the mechanisms underlying long‐term clinical improvement remain unclear. In the present study, the beneficial effects observed at day 30 may be more cautiously interpreted as the result of enhanced oral sensory awareness, facilitation of swallowing initiation, and increased attention to safe swallowing behaviors before meals rather than direct evidence of long‐term neural plasticity or structural cortical changes. These findings support the beneficial effects of sour taste as an oral stimulus in improving swallowing function and preventing complications in acute ischemic stroke patients. The study underscores the clinical relevance of administering sour taste as a practical nursing intervention to enhance patient care quality in the acute post‐stroke period.

The NIHSS is widely used to evaluate neurological status. Although scores may initially be zero following acute stroke, patients may experience subsequent motor deficits, with NIHSS scores changing over time (Keheya et al.^2016^; Hand et al. 2014). In this study, mean NIHSS scores were analyzed at the initial assessment, day 7, and day 30, showing expected reductions over time consistent with the natural course of recovery. Okubo et al. (2012) demonstrated that NIHSS is highly sensitive (88%) and specific (85%) in detecting swallowing disorders after acute ischemic stroke, and Henke et al. (2017) confirmed its reliability for identifying changes in swallowing function (Henke et al. 2017). A significant negative correlation between NIHSS and GUSS scores was observed in both groups, indicating improved swallowing function with reduced stroke severity. These results are consistent with previous literature.

Based on these findings, it is recommended that swallowing function in the early post‐stroke period be assessed with a comprehensive bedside evaluation and that sour taste, such as lemon juice, be administered before meals. Therefore, sour taste stimulation may be considered a simple, non‐invasive, feasible, and low‐cost complementary sensory intervention that can be integrated into interdisciplinary dysphagia management in clinically stable stroke patients. Nevertheless, several methodological limitations should be considered. First, complete participant blinding was not feasible because a sour taste could be recognized by participants receiving lemon juice, which may have increased the risk of performance bias. Second, analyses were conducted using a per‐protocol approach because participants who died during follow‐up could not complete outcome assessments. Third, the study was conducted at a single center, which may limit the generalizability of the findings to different clinical settings and patient populations. In addition, the follow‐up period was limited to 30 days, and therefore the long‐term sustainability of the intervention effects remains unclear. Future studies using instrumental swallowing assessments such as VFSS or FEES and longer follow‐up periods are needed to clarify the physiological mechanisms and long‐term clinical effects of sour taste stimulation. Future research should also investigate the effects of other taste stimuli, such as bitter and sweet, and evaluate combined rehabilitation approaches within an interdisciplinary framework.

## 5 Conclusion

This study demonstrated that administration of a sour taste to patients who developed dysphagia during the early period of acute ischemic stroke had a beneficial effect on swallowing function. The findings suggest that sour taste stimulation may serve as a practical, feasible, and complementary sensory intervention to support swallowing function in clinically stable stroke patients. The observed improvement in swallowing function may be associated with enhanced sensory stimulation and facilitation of swallowing initiation rather than direct evidence of neural plasticity or structural brain changes. These findings may contribute to the development of supportive rehabilitation strategies for post‐stroke dysphagia management. From a nursing perspective, its implementation should be accompanied by careful monitoring for signs of aspiration, including coughing, throat clearing, wet voice quality, oxygen desaturation, or respiratory distress. If these signs occur, oral intake should be discontinued and swallowing function should be reassessed before resuming feeding.

### 5.1 Study Limitations

Nevertheless, some methodological limitations should be considered, including incomplete participant blinding, the single‐center design, the absence of instrumental swallowing assessments, and the relatively short follow‐up duration. Therefore, the findings should be interpreted cautiously and confirmed in larger multicenter studies using objective swallowing assessments.

## Author Contributions

**Ayfer Gunes**: conceptualization, methodology, writing – original draft, writing – review and editing. **Ozgul Erol**: conceptualization, methodology, writing – original draft, writing – review and editing, and supervision. **Sezgin Kehaya**: conceptualization, methodology, formal analysis, writing – review and editing, and supervision.

## Declaration of Generative AI and AI‐Assisted Technologies in the Writing Process

During the preparation of this work, the authors used [DeepL, Gemini] in order to improve language readability. After using this service, the authors reviewed and edited the content as needed and took full responsibility for the content of the publication.

## Funding

The authors have nothing to report.

## Ethics Statement

Ethical approval was obtained from the scientific research ethics committee of a university, with decision number 13/32 dated 14.06.2021 and protocol number TÜTF‐BAEK 2021/214. Institutional permission was also obtained from the clinic where the research was conducted. After obtaining the necessary permissions and ethical approval, the informed consent form was presented to the patients and their relatives, and their verbal and written consent regarding the research was obtained. It was clearly stated that the research data would be used only for scientific purposes. The implementation of the research adhered to the Helsinki Declaration, Good Clinical Practice Guidelines, and TÜTF‐BAEK Directive.

## Conflicts of Interest

The authors declare no potential conflicts of interest with respect to the research, authorship, and/or publication of this article.

## Data Availability

The data that support the findings of this study are available from the corresponding author upon reasonable request. The data are not publicly available because they contain information that could compromise the privacy of the research participants.
